# Development of an on-job mentorship programme to improve nursing experience for enhanced patient experience of compassionate care

**DOI:** 10.1186/s12912-021-00682-4

**Published:** 2021-09-18

**Authors:** Alma Arshad Hookmani, Naureen Lalani, Noureen Sultan, Aly Zubairi, Ayesha Hussain, Babar S. Hasan, Muneera A. Rasheed

**Affiliations:** 1 Aga Khan University, Karachi, Pakistan; 2 Aga Khan University Hospital, Karachi, Pakistan; 3 Charter for Compassion, Karachi, Pakistan; 4 Center for International Health, Department of Global Health and Primary Care, University of Bergen, 5700 Bergen, Norway

**Keywords:** Compassion, Nursing, On-job mentorship, Pakistan, Patient experience, Theory of change

## Abstract

**Background:**

Evidence suggests improvement in nursing staff satisfaction, competence, and retention after implementation of evidence-based mentorship programmes. When guided by a framework of compassion, mentoring as a caring action can not only build healthy, transformative relationships but a similar behavior is reciprocated to patients which subsequently can drive patient experience of care. However, examples of on-job mentorship programs for nurses in low- and middle-income countries (LMIC) are limited.

**Objective:**

The objective of the study was to develop an on-job nursing mentorship programme using a compassionate framework aimed at improving nurses’ experience and thus enhancing patient experience in a tertiary care hospital in Pakistan.

**Methods:**

Designed as an intervention development study, it was completed between January 2018–December 2019. The programme was developed by a team composed of service and nursing leadership, director patient experience of care and a compassion specialist using a theory of change model. The package followed a series of steps, a) identification of a framework, b) creation of working group c) needs assessment and d) multiple meetings to frame the model followed by implementing the preconditions for roll-out of the programme with the frontline staff.

**Results:**

The eventual outcome was improving the patient’s experience of compassion while the intermediate outcome was to have nurses demonstrate compassionate care. The pre-conditions were identified as: recruitment of staff with appropriate skills for pediatric care, provision of compassionate experience to the frontline nurses by addressing their specific pain points, development of competent head nurses as supervisors and creation of a compassionate culture. To ensure the pre-conditions, various interventions were planned with some implemented through the course of the study while others are in the process of being rolled out. These involved, inclusion of pediatric compassion specific module during orientation of new hires, creation of space to talk about compassionate skills with staff, provision of trainings and mentorship to create competent head nurses, and creating a culture that promoted and recognized compassionate care values.

**Conclusion:**

The approach helped to delineate feasible pathways for an on-job compassionate mentorship programme enhancing routine supervisors' role as facilitators of compassionate care.

**Supplementary Information:**

The online version contains supplementary material available at 10.1186/s12912-021-00682-4.

## Background

Patient-centeredness defined as ‘providing care that is respectful of and responsive to, individual patient preferences, needs and values, and ensuring that patient values guide all clinical decisions’ by the Institute of Medicine has lately been acknowledged as an essence to best quality services [1]. The implementation of patient-centeredness also requires direct interaction and a strong relation with the patient which mostly happens through nurses. Owing to the characteristic frequency of nurse-patient interactions, they naturally emerge as a gateway for improving patient experience. The nurse-patient relation is strong to such an extent that the practice nurses become a central point in shaping and improving patients’ experience [2]. Hence, apart from possessing the knowledge and technical skills, demonstrating compassionate behaviors is essential to tackle circumstances [3], fulfill patients’ needs, and improve not just the patients’ but also their own well-being [4]. A pilot intervention study with nurses utilizing self-compassion training showed reduced burn-out scores while improving scores on resilience and compassion fatigue with an effect size of 1.23 [5], Another study utilizing a similar training for self-compassion working with 64 nurses in three university hospitals in Ireland found significant reduction in perceived stress after a 6-week training [6]. Further, nurses’ well-being has been indicated to be mediating the relationship between health services and patients’ experience of care (indirect effect = 0.014; 95% Confidence Intervals = 0.002–0.036) in a study with a sample of 173 nurses and 3525 patients [7]. A review focusing on training meant to enhance compassion linked greater healing and health outcomes to improved patient-centered communication by the providers [8, 9].

Bearing such significant responsibility and engaging compassionately is surely rewarding but exhausting as well [10]. It comes with certain consequences, one of which is compassion fatigue. However, it is not only caused by the prior but also from regular and prolonged encounters with clients [11] and situations in work where nurses feel helpless [12]. The fundamental urgency ‘to do something’ [13] and to eliminate others’ suffering contributes to the distress, enabling self-dehumanizing tendencies, which commonly strain one’s relationship with close ones.

Another effect faced by these nurses is burnout, which descends from compassion fatigue [14], but it occurs when a person is unable to achieve his/ her targets causing frustration, loss of control [15] subsequently effecting patient safety, clinicians’ health and workforce retention [14]. The top two reasons for compassion fatigue are less administrative support and lack of satisfaction with colleagues [16]. In order to avoid compromising on patient experience, nurses should be trained to be compassion literates [15] by focusing on internal motivation, nurturing self-compassion, increasing staff engagement, and promoting compassionate culture and leadership [17]. However, few programs (e.g., precepting and internships) with aims to enhance compassion literacy among nurses [18], have unfortunately been ineffective due to certain disadvantages i.e., time and administrative constraints, occasional absences of preceptors, interrupted continuity and discrepancies, and lack of evidence for cost effectiveness thus adding to sustainability concerns [19]. Since compassion is a virtuous cycle that extends from assistance of superiors to care delivery of patients in healthcare [20], leadership by the superiors, precisely through on-job mentorship, is considered as a means for generating sustainable benefits in the field of nursing [21].

Mentoring is an interpersonal phenomenon [22] that aims to nurture the well-being of the novice through both informal and formal encounters [23–25]. Such encounters can be in the form of supporting, guiding, teaching, role modeling, counseling and sharing [26]. Even though the formal nursing mentorship programs started in the late 1970s [25]; however, there has been a lack of focus on building nursing mentorship models [23] and insufficient documentation has been done on mentorship environments present for them [26]. Organizing mentorship programs is crucial as they improve retention rates, enhance job satisfaction [27, 28] and reduce compassion fatigue [29]. Moreover, confidence, competence, and growth also improve due to the mentoring relationship [30].

It is not just the nurses but patients who have also advocated the need for a support system to sustain compassion in the nursing practice [31]. The managers have been identified to be well-placed in this role of mentors given the nature of their relationship with frontline staff. Embedding compassion in the system to be adopted by nurse managers as mentors can be a promising undertaking to teach nurses compassion or as cogently coined by Burridge et al. [32], ‘compassion literacy’. A compassionate nursing leader plays a critical role here in filling the void and creating a compassionate culture for nurses, who experience compassion vacuum on the job [33].

A recent study in a private hospital in Pakistan reported an increase on nursing indicators 18–27 months post-intervention as part of their patient experience initiative [34]. The improvement was attributed to implementation of an on-job mentorship programme. Since little has been written on how to develop, implement, and sustain nursing compassion for improved patient experience outcomes in low- and middle - income countries (LMICs), the current study aims s to describe the development of the on-job mentorship programme designed to provide compassionate experience to frontline nursing at a systemic level subsequently leading to enhance patient experience of care. The paper specifically aims to propose mentoring as an efficient technique to develop compassion focused approaches among on-job nurses. However, it is not just this, but the study also highlights mentoring being a type of micromanagement that can empower nursing staff.

## Methods

### Setting

Established in 1985, Aga Khan University Hospital (AKUH, K) is located in Karachi, the largest city of Pakistan and serves two provinces, Sindh and Balochistan, with populations of 47.89 million and 12.34 million, respectively. It is a tertiary care hospital with consultant doctors specialized to provide optimal care and treatment to patients with a full range and severity of specialty diseases and conditions. Recently, Patient Experience is one of the six approaches among others, including Community and Family Health and Patient Safety and Quality adopted by AKUH, K to facilitate collaborative operations where physicians, nurses, researchers, and teachers work in unison to give patients the best access to care. AKUH, K is also Joint Commission International accredited which focuses on patient safety. Through its advanced resources, trained faculty, and technological aid, the institute is creating models for research projects rendering short and long-term impact in the provinces. Paediatric service is the largest service line of the hospital having 122 inpatient beds and has the largest number of the nursing services employees (*n* = 413) providing high quality, multi-specialty care. All major medical and surgical specialties are present to deliver excellent pediatric care for complex health problems. The nursing management team in the service line consisted of a nurse manager as the senior position followed by a specialist, head nurse and then frontline nursing staff (Supplementary Figure 1). The study was approved for an exemption as Quality Improvement (QI) study by the Ethics Review Committee of the Aga Khan University and as per institutional requirements consent was waived (being an exemption).

### Design of the intervention

Designed as an intervention development study [35], this current programme was part of a larger quality improvement initiative to improve patient experience and was conducted between January 2018 to December 2020 [34]. The Theory of Change (ToC) model was utilized to develop a solution to nurture engagement among nurses, facilitate relationship building and impart soft skills thus instilling a satisfactory employee experience for subsequent improved patient experience. ToC has been widely used and it is considered to be the basis of monitoring and [36] theory-based evaluations [37–40]. According to Weiss [41], ToC is, “a theory of how and why an initiative works”. A robust ToC embodies several elements that makes the model more systematic [36].

The nursing mentorship ToC comprised preconditions, interventions, outcomes and the final goal. The final impact was to achieve improved compassionate experience of patients through refined skills of compassion in nurses. The preconditions are a necessary requirement, condition or element that should be acknowledged for achieving the desired outcome while to fulfill these preconditions or remove dodges, it is important to have efficient interventions that create a positive difference in outcomes and impact of interest [36].

### Development of the intervention ToC model

The development of the model took several steps as described below (Table 1).

**Table 1:** Steps for developing the Theory of Change model

Aim	Method	Output
To conceptualize the problem from the perspective of external customer	Patient feedback survey and subsequent meetings with the leadership	It was realized that parents’ felt that the staff was disengaged, and their top recommendation was to improve responsiveness and communication to meet emotional and informational needs.
To conceptualize the problem from the perspective of internal customers	Survey and thematic analysis	Top pain points were identified that affected their ability to provide compassionate care: perception of being overburdened, poor connection with work and lack of growth opportunities.
To identify a conceptual framework for evidence-based package	Literature review of mentorship packages available for nurses	Identification of ‘Caring Mentorship Model’ for application in the service.
To assign a group of individuals committed to the design of package	Meetings with service and nursing leadership to identify individuals with strengths to inform the package.	Individuals identified with strengths across: human experience, health outcomes, nursing management, compassion and implementation sciences.
To experience the barriers the staff and supervisors face when delivering patient care and employee care.	Work shadow of frontline staff, discussion on departmental Facebook page, and discussion with the nursing working group	Barriers to patient experience included: 1. Inefficient inter services coordination
		Barriers to employee experience included: 1. High staff-supervisor ratio (1:60) 2. Unequal (or no) access to basic facilities
To identify preconditions and design interventions accordingly.	Regular working group meetings	The following overarching pre-conditions were identified: 1. Hire staff with the right skills for pediatric care 2. Define compassionate experience 3. Have competent head nurses as supervisors 4. Create a culture of compassionate experience
		The following interventions were agreed on: 1. Include paediatric compassion specific nursing education service (NES) orientation program 2. Communicate about compassionate skills, revise career ladder and provide trainings and on-job coaching 3. Strengthen supervision, conduct workshops create checklists and develop dashboards for data visualization 4. Promote compassionate values and recognize high-performing nurses

#### Conceptualization

As the service leadership and director of patient experience of care sought to work on improving patient experience in the service line, a survey was conducted between July–August 2017 with families in the paediatric ward to determine the issues that affected their experience using the Child Health Consumer Assessment of Providers and Systems (HCAHPS) [42]. The form also included items on care from nurses. The survey was completed with 221 families admitted for at least 24 h and age between both to 6 years. The overall results have been reported in another manuscript [34]. Findings around care from nursing items indicated that 75% of patients reported that nurses always communicated with courtesy and respect, 71% said nurses listened to them carefully and 77% felt nurses explained things in a way they could understand. Also, only 27% reported getting help from staff when they pressed the call bell. There was also an open-ended item requesting families to list down the top three factors that affected their experience during stay. About 38% indicated lack of responsiveness from nurses followed by 29% stating ineffective communication. This led to conceptualization of the problem that the staff was disengaged and an intervention to improve patient experience meant engaging the staff via improving their own experience through a ‘compassionate mentorship package’ integrated into their existing supervision system.

#### Identification of a framework

After a literature review, the core team identified the Caring Mentorship Model [43] as a conceptual framework to address the phenomenon of building relationships through mentorship of nursing staff. This model has developed from two other models of the Development of Caring Nurse-Self  [44, 45]. It depicts three phases: relationship with no connection, surface connection, and shared connection with each containing three levels that a person reflects at: cognitive (task-oriented mentoring), affective (interactive mentoring), and transformative (transformative mentoring). The programme intended to work on encouraging *supervisors* to become attuned to *mentorship* roles. The goal of the programme was to enable the head nurses to form a meaningful and reflective relationship with the nurses beyond the cognitive and interactive understanding to improve employee experience leading to enhanced patient experience. Table 2 states all the questions addressed in accordance with identified model [43] and the activities incorporated by the nursing mentorship programme in each phase.

**Table 2:** The questions addressed and activities in the mentoring phases and levels of Wagner’s model

Phase and Level	Addressed Questions and Activities
Relationship with No Connection - Cognitive (Task-oriented Mentoring)	Understanding is limited to, ‘What is happening?’ or ‘What are the details?’. In this phase, the activities focus on technical and coordination skills, ensuring support to the nurses, ensuring attendance and working on organizational skills.
Surface Connection - Affective (Interactive Mentoring)	Reflecting questions like, ‘What is important here?’, ‘What am I feeling?’ or ‘Who am I?’ (Wagner, 2005a). The manual emphasizes communication skills, encouraging positive interactions, ethics and completing patient notes while reflecting on patient sociodemographic details that can affect the treatment outcome.
Shared Connection - Transformative (Transformative Mentoring**)**	The mentors reflect on questions that cater to self and his/ her mentees. These questions include, ‘What relationship do I see?’, ‘What is the meaning?’ or ‘What more is possible?’. The activities here include a focus on building deeper meaning in relationships and enhancing interactions with the patients e.g., through use of play.

The team was unable to formally evaluate where the nursing staff stands in terms of mentorship phases. The informal team reflections about the baseline assumption suggests the connection was at the cognitive level with surface connection. There was no human connection and mentorship being limited to only monitoring and completing checklists. Hence, the aim was to create opportunities and provide a safe place for the connection to evolve at an ‘affective’ and later ‘transformative’ level. It was important to create a humane culture for nurses to reciprocate a similar habitat for patients.

#### Creation of a working group

The next step was identifying a well-organized team with the right skills that was designated to work on this task. The service leadership identified the working group to lead the change effort (Supplementary Table 1). Along with the Service Line Chief and Director Patient Experience, members included Nurse Manager, three Nursing specialists, and a Research Specialist. While the internal group developed technical solutions, external expertise was needed to solidify the compassion angle. Therefore, the team reached out to Charter for Compassion Pakistan (henceforth, CfC Pakistan – a local initiative inspired by the Charter for Compassion International) and through shared learning of organizational behavior change and nine skill- based compassionate package, respectively, set in motion a mutually beneficial partnership. The team members from CfC Pakistan included a project lead and an associate.

#### Meetings of the working group

A regular schedule for meetings was carried out to co-design the package. The team members met once a week for the initial 4 months moving to once every 15 days in the next 2 months. The meeting was conducted during working hours every Friday and typically lasted for an hour. Each meeting held discussions on how to achieve the month’s agenda and meeting minutes were recorded by the nursing lead. Certain ground rules were set for these meetings which involved application of an empathetic approach, clarity of roles and responsibilities, valuing inputs from all based on their strengths and focus on outcomes and feasibility.

The tasks of the meeting group for design of the implementation of the interventions (frequency, dosage, timing) specifically were divided according to the RACI matrix (Responsible, Accountable, Consulted and Informed). This matrix proves to be beneficial in organizational context as it allows to identify the roles anticipated for each person and any missing roles for early corrections [46]. The explanation of each component is as follows:

1. Responsible: The member under this section had the responsibility of completing the task. In the current study, e.g., the tasks of designing supervisory checklists and Nursing Education Services (NES) pediatric modules were assigned to a nursing specialist (NS) who was already performing such duties. She was responsible and owned the whole course of action.
2. Accountable: The nursing manager (NL) was accountable to have the final approving authority for the work around supervisory checklists and designing of NES paediatric modules completed by the respective members. Contrary to the ‘responsible’ team, the accountable one was answerable for the task.
3. Consulted: The consultants i.e., director patient experience of care, (MR), chief of Service Line (BH) and compassion specialist (AZ), provided valuable input on the relevant tasks by engaging in two-way communication. They brought in different perspectives regarding pre-conditions and designing modules.
4. Informed: The higher leadership roles i.e., the Chief Nursing Officer, Dean of School of Nursing and Midwifery, CEO and others belonged to this group. They did not provide any input, rather kept a track of needs, preconditions and assessments and developments of interventions.

A systematic process was adopted to design the model. The first step was understanding the contextual needs and then framing the pre-conditions followed by developing indicators to assess the effectiveness of the model. Feasibility was the key consideration of designing the interventions [47]. A few principles around different components of feasibility were agreed upon by the working team to guide the process (Table 3).

**Table 3:** Key areas of focus for feasibility of the interventions

Areas of Focus	Questions
Acceptability	What is the core issue leading to suboptimal patient experience? What can be done to eliminate it and prevent it?
Demand	How much demand is likely to exist for compassionate on-job mentorship programmes?
Implementation	Who will execute it? When will it be appropriate to conduct the intervention and who will follow-up with it?
Practicality	Do we have relevant human and financial resources? Is the method feasible?
Adaptation	Can the compassionate on-job mentorship programme be applied to the adult-service lines in the hospital?
Integration	Does the task align with the respective individual’s job description? How can compassionate care skills checklists be integrated in the existing performance system.
Limited-efficacy testing	Will the mentorship process be able to encourage nurses to demonstrate compassionate behaviors?

#### Needs assessment

##### Pain points survey

A brief survey was conducted to understand the factors that affected the engagement of the nurses. It included 3 items around factors that affected them personally, the patients and overall service delivery. The survey was meant to be shared by the nursing supervisors with all the nurses as a paper-form and to be completed anonymously. However, only 173 (42%) forms were returned. One reason was that the managers were not able to share the form with staff on night duties. Moreover, the staff was spread across 11 different locations and not all of them would have received on time. Gathering nurses, distributing the form, and then collecting it was a considerable effort. Given the urgency of the matter, the team utilized the forms received for the analysis. The working team also did not want to push harder for additional forms lest the staff should feel coerced.

A compiled list of 329 pain points was received from the nursing service coordinator. A thematic analysis following an inductive approach [48] was completed by a behaviour implementation scientist (MR) and a psychology graduate (AAH). The pain points were independently coded followed by agreement of the codes in a face-to-face meeting. The analysis revealed three main themes: perception of being overburdened, lack of connection with work, and lack of growth opportunities. Perception *o*f being overburdened meant that the nurses viewed their work as being excessive with prolonged procedure, night shifts, call on off days, high expectations or pressure from management, excessive documentation etc. While lack of connection with work holds two main themes: accountability and lack of facilities. Lack of facilities included low salary, breaktime issues, one washroom for 30 staff, no medical benefits for their parents, unavailability of instruments, etc. However, accountability catered to different issues that according to the nurses were ethically inappropriate. These involved over criticism from seniors, revenge seeking behaviors of co-workers, work environment, and many more. The last pain point i.e., lack of growth opportunities comprised concerns like insecurity of jobs, no promotion of registered nurses (RNs) and no future for the more senior, older staff. As per the answers, ‘Perception of being overburdened’ was found to be the most common theme among the responses. The findings from the thematic analysis were shared with the nursing manager and supervisors as a means to establish trustworthiness of the findings.

##### Review of existing documents

Like NES modules, nursing competency checklists led to the realization that few shortcomings had to be addressed to work on patient-centeredness and achieve the final outcome. The existing supervision or performance management system aimed to assess nurses every 6 months based on a checklist for hard skills only. There was also no training for head nurses (supervisors of frontline nurses) to enhance their supervision skills. The NES modules were designed for adults only with no integration of pediatric skills which meant the newly hired nurses had to be retrained once they joined Pediatric service.

##### Work shadow

After the discovery of pain points faced by the nursing staff, it was necessary to identify the workload of nurses during their shift leading to devise appropriate strategies to increase satisfaction and boosting effective service. Catering to this purpose, it was important to implement an in-depth direct observation to gain a comprehensive understanding of the current settings. Hence, the research team used ‘shadowing’, which is a technique that involves a researcher following a member of the organization meticulously, over a length of time [49].

In February 2018, a bed-side nurse, a head nurse (administrative supervisor) and an instructor head nurse (clinical supervisor) were shadowed, during his/ her work shift by a research associate. According to the observations, the bed-side nurse spent most i.e. 25% of her time, caring for patients and addressing their complaints, while patient shifting took the least time. The instructor head nurse spent most of her time on nurses’ education planning (23%, 71 min), and the administrative head nurse spent most time in rounds i.e., 36% (111 min). Challenges faced by the nursing staff were identified, which were categorized as inter service coordination. These included, difficulties in getting their needs fulfilled like lack of lunch facilitation or unavailability of medicines in pharmacy during night. Furthermore, it was revealed that the staff-supervisor ratio was too high, each supervisor (administrative and clinical) had to oversee more than 64 nurses. Both the supervisors were looking after the same staff but for different reasons (clinical skills and administration). In order to overcome some of these issues it was suggested to promote equality in terms of providing meals and being treated with respect like other cadres e.g., trainee physicians or provision of equal facilities e.g., having a shared restroom. Another recommendation was to make one supervisor (head nurse) responsible for both academic and clinical responsibilities for 30 nurses reducing the burden given both roles required similar credentials. So, one head nurse is whole sole responsible for the academic and clinical growth of their nursing staff. The mantra was to be vigilant and responsive to the workforce i.e., nurses’ needs. Once they feel that they are being taken care of, they will resonate a similar attitude towards patients and bring in the best patient care to the healthcare organizations.

## Results

**Fig. 1:**
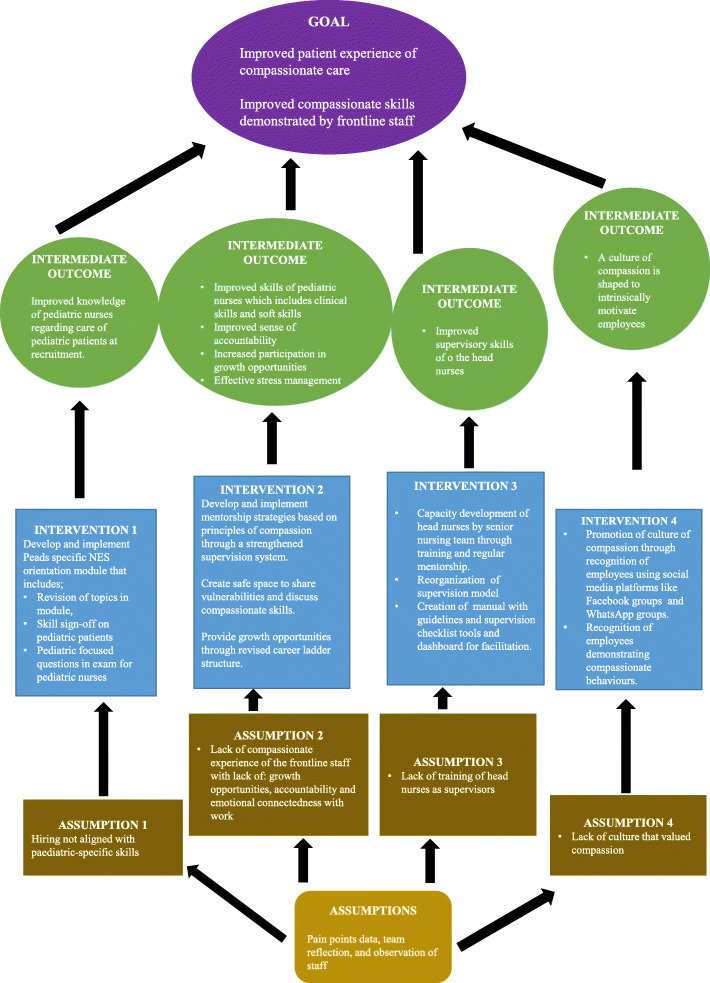
Theory of Change (ToC) hinged on integrating compassionate mentorship for nurses into routine supervision

The results section describes the ToC model created including the goal, the pathways to change and the interventions to enable the processes leading to the pathways. The model helped to first identify the desired long-term goals and the members worked backwards from these to identify all conditions (outcomes) that must be in place for the goal to occur. Later, all of these were mapped out in an outcome framework (Table 4 and Fig. 1). Correspondingly, the model for nursing mentorship program was developed incorporating the following:

**Table 4:** The Theory of Change model

Impact: Improved compassionate experience of patients (in progress) N (%) patients rating experience with nursing staff as excellent			
Intermediate outcome: Improved skills of compassion in nurses N (%) nurses rated as excellent, good, average and poor by the respective head nurses			
Pre-conditions	Interventions	Indicator/s	Status
1. Hiring staff with the right skills for pediatric care	Include paediatric compassion specific modules in the nursing education service (NES) orientation program	N (%) nurses trained for pediatric specific compassionate skills per batch	Completed
2. Provision of compassionate experience to the frontline staff defined as			
2.1 Feeling of emotional connectedness with work	Create space to talk about compassionate skills with employees	Attendance rates in the workshops Attrition rates	Not yet
2.2 Formal and informal on-job training around compassionate behaviours	Provide trainings Provide on-job coaching through formal observation and feedback	N (%) nurses trained annually N (%) nurses observed N (%) nurses rated as excellent	Not yet Not yet
2.3 Fair growth opportunities	Revise career ladder of supervisor to be appraised on mentee outcomes	N (%) attended a professional growth activity every quarter N (%) scholarly outputs every quarter	Initiated
3. Competent head nurses as supervisors			
3.1 Training of supervisors as compassionate mentors	Conduct regular training workshops	N (%) supervisor trained annually	Completed
3.2 Availability of supervisory tools	Creation of checklists Developing dashboards	N (%) of eligible checklists completed N times dashboard accessed on a monthly basis per supervisor	Initiated Initiated
4. A culture of valuing compassionate experience	Recognition in town hall meetings, WhatsApp groups and morning huddles	N (%) nursing of total appreciation per month	Initiated
	Recognition of nurses by the patients counted for performance appraisal.	N (%) patient rating nurses as excellent	Initiated
	Promotion of compassionate values on department Facebook page	N (%) posts from nursing staff per month	Initiated

### Impact

The analysis of patient feedback forms had shown an inadequate responsiveness to patients’ needs, due to less engaged workforce. Hence, the impact of all the interventions was agreed to be an improved compassionate experience of patients, provided by the nurses. The indicator for measuring impact was N (%) patients rating experience with nursing staff as excellent as measured by Child HCAHPS.

### Intermediate outcomes

A better employee experience will ultimately enhance patient experience therefore, hence it was ensured to include mentorship of nurses as a core intervention strategy. The intermediate goal included improvement in compassionate skills of nursing staff as indexed by supervisors’ (head nurses) rating N (%) nurses on performance (good, average and poor) using the checklists. The interventions will be measured using separate indicators and based on this, necessary changes will be proposed. The success of the NES orientation program will be measured depending on the number (%) of nurses trained for pediatric specific compassionate skills per batch. In order to ensure widespread compassionate experience in every unit, attendance rates in the workshops, attrition rates were taken into consideration. It is also planned to use a number (%) of nurses trained annually and observed during formal on-job observation during patient care activities, for the said purpose however, it has not been initiated as of yet. Moreover, the outcomes of supervisors’ revised career ladder would be measured by reviewing the number (%) of supervisors who attended a professional growth activity and number (%) of scholarly outputs every quarter.

After training and providing tools to supervisors, its outcome was planned to be measured by observing the number (%) of supervisors being trained annually, checklists completed and times the dashboard was accessed by a supervisor on a monthly basis. Moreover, the effects of creating groups on communication platforms to increase social engagement was measured through the number (%) of total appreciation for nurses from peers and other health care professionals and posts from nursing staff per month. Lastly, the performance appraisals will be provided based on receiving ‘excellent’ ratings on patient feedback form and soft skills checklist.

### Pre-conditions

Several pre-conditions were identified in the paediatric service line to achieve the goal of improved patient experience. These were categorized as:

1. Recruit staff with the right skills to achieve the ultimate pediatric care. According to many employers, the issue arises when the required skills are short in supply [50, 51]. Since the patient needs are evolving, it is necessary for the workforce to be less rigid and adopt new directions to fulfill the needs through ‘broad-based’ training and skills [52].
2. Build a compassionate experience to address employee pain points to enhance their engagement as defined by the nursing staff on the survey: feeling of emotional connectedness with work, formal and informal on-job training for compassion and fair growth opportunities. These pre-conditions also serve to address the perception of being over-burdened.
3. Maintain or increase competency of head nurses as supervisors by training them as compassionate mentors and making supervisory tools accessible. Mentoring itself is a convoluted process that requires the supervisors to provide constructive feedback and counselling, reflect and encourage and model good teaching practice [53].
4. Create a culture of compassionate experience through instant communication, emulation of compassionate principles and acknowledgment of the staff’s vigorous efforts in providing adequate care. The team employed multiple platforms including social media to impart the head nurses (both administrative and clinical supervisors) with a sense of meaning and promote organizational citizenship behavior. Association of public social communication with risk and ethical issues (privacy, productivity, training and education) has made organizations and the healthcare industry reluctant to adopt social media as a means of communication [54], however, recent evidence states otherwise, especially when work is majorly virtual in the face of pandemic [55].

### Interventions

This section explains the specific interventions designed for the corresponding precondition. The completed or ongoing interventions were around revising requirements to hire staff for the right skills, preparing the head nurses (nurse supervisors) through training and provision of supervisory tools and promoting culture change which were part of the larger initiative. The roll-out with the frontline staff was delayed due to the pandemic crisis.

#### Hiring with the right skills

At AKUH, the NES department conducts basic orientation for all newly hired nurses, at the time of induction. The focus is on basic theoretical and skill competencies required at entry level for safe care provision to adult patients. The core team collaborated with the NES department to revise orientation and included paediatric specific modules along with an introduction to principles of compassion. Orientation to compassionate skills ensured that the nurses were not only trained for hard skills pertaining to the pediatric population but were also encouraged to learn soft skills as well, so that compassionate skill set was imparted to the new nurses before they started patient care. Following the initial roll out, refresher sessions were also planned. The intervention was proposed to lead to two main outcomes: improvement in nurses’ knowledge and skills regarding care of pediatric patients and skills and compassionate interactions with the patients and their families.

#### Experiencing compassion at workplace

It was also aimed to create a compassionate experience for the staff through various ways i.e., creating a safe space where nurses could share their vulnerabilities as staff and also as individuals, providing opportunities of growth through formal and informal training and clarity of growth trajectory while revising the career ladder. However, only the last aspect could be initiated due to the pandemic crisis in the hospital.

##### Feelings of emotional connectedness with work

As part of the on-job training, one-hour teaching sessions were conducted regularly for the nursing staff by their academic supervisor. The sessions were usually designed for at least 5 days a week and a monthly planner was developed by 22nd of each month. These sessions were planned for fulfilling nurses’ academic needs or building their clinical skills. As part of the programme, one session per week was designated for compassionate skills to be discussed each week. The presentations and materials were prepared by the head nurses (Supplementary Table 2) through an extensive exercise with support from CfC.

##### Formal and informal on-job training for compassionate skills

Feng et al. [56] found that out of 10 items, 2 (child comfort and nurse-parent communication) were consistently and strongly associated with willingness-to-recommend scores. Hence, it was important to have these skills integrated into on-going on-job coaching and supervision interactions to identify teaching moments. On-job coaching was conducted with the bedside nurses during daily rounds. The head nurse observed nurses at work and were now required to provide feedback based on the principles of mentorship. The principles were being available for the staff, listening to them, taking responsibility and giving credit to their mentees, inspiring and build a transparent relationship with them with a focus on personal and professional development.

##### Growth opportunities

The career ladder and promotion criteria for head nurses and their supervisors i.e., nursing specialists was revised to address the nurses’ and their own needs for professional growth and development opportunities. This implied that promotion of the supervisors and compensation would now be based on the outcome achieved for the supervisees i.e., prime patient care, nurse’s experience and credentialing. The minimum requirements for senior positions such as ‘Head Nurse’ or ‘Nursing Specialist’ (supervisors of head nurses) were updated to 4–5 and 6–8 years of experience respectively (Supplementary Figures 2 and 3). Moreover, receiving compassion skills training and experience in research and publication along with specific certifications such as ‘Basic Life Support’, ‘Safe Medication Administration’, ‘Conscious Sedation’ etc. were made compulsory for supervisory roles. With respect to mentoring, these positions were also required to serve as a role model for other staff, to conduct needs analysis of unit nurse instructors and to identify staff learning needs, to develop and implement appropriate teaching interventions and other duties that would ensure cost effective patient care outcome.

#### Strengthened supervision system

##### Trainings of senior nursing team (nursing specialists)

A training workshop was planned and conducted by Compassion in Healthcare (CiH) lead from CfC Pakistan, for the senior nursing team including nursing specialists (supervisors of head nurses) and head nurses (supervisors of the bedside nurses) during June and July 2018. These workshops aimed to create opportunities for self-reflection by cultivating an understanding of the principles of compassion, namely: self-compassion, mindfulness, empathy, gratitude, forgiveness, courage, integrity, and altruism-which formed the building blocks of mentorship. This training workshop was created by CfC Pakistan after several visits in all the wards where discussions about the challenges and adversities took place with the on-call nurses and doctors. Supplementing the training workshop on soft skills, i.e., compassion and communication with supervisees (mentees), another workshop was piloted with a focus on hard skills, i.e., the effective supervision tools i.e., learning to observe, providing feedback, problem-solving and analyzing data (Supplementary Table 3). Following this, a final session was conducted to summarize the overall content through quiz and team discussions. Post training feedback was taken from the participants to ensure they understood the practices that should be implemented for the betterment of nursing experience. A few participants stated in their training evaluation that the training helped them to realize the significance of compassionate skills for their supervisees: *“It made me realize to focus on small things in life by showing gratitude, empathy and self-compassion and so on. I think this training will help to ripple the magic of kindness in the whole institution.”* Another head nurses felt the training was engaging and allowed them to reflect*. “Very engaging and gave a platform for self-reflection … helped us to reflect on ourselves.”*

##### Mentorship of the head nurses by the senior nursing team

To ensure the sustainability of the mentorship skills demonstrated by the head nurses (supervisors of nursing staff), another layer of mentorship was included specifically designed to be received from their own supervisors i.e., nursing specialists (supervisors of head nurses). A standard operating manual was developed for them stated that the specific goals for the head nurses should be created in alignment of skills building of the frontline nurses. The main dimension of supervisory roles and responsibilities included leadership skills with intended outcome of talent retention and transfer of key knowledge to the head nurses and nurses and improving and strengthening culture of compassion. Other responsibilities when mentoring head nurses included measuring and categorizing reflections on a scale of absolute to independent reasoning, ensuring that the reflections were progressing according to the month’s plan, evaluating head nurses on the basis of competency or value, assessing the skills practices in a day, respecting their personal details and vulnerabilities, finding solutions to eradicate the stress points, and sharing their views about what they think of their own mentees (bedside nurses). The reflections were not limited to work life; they aimed to gather variables from both personal and professional lives to assess correlations that create both meaningful and toxic results.

A supervisory checklist was designed to observe and rate the performance of the head nurses to facilitate skills-building for mentorship (Supplementary Table 4). The head nurses were observed (by nurse specialist) on the basis of five skills with each comprising different responsibilities upon which they were rated; organizational (e.g. punctuality and regularity), technical (observes the staff and provides on job coaching effectively), education (maintains pace during sessions and encourages participation), communication (encourages, praises the staff and also maintains privacy of their performance), and coordination (teamwork and positive attitude). The checklist consisted of a three-point Likert scale: 0 being ‘no skill’ to 3 being ‘performance with excellency’. The skills were rated, and a total score was calculated by adding all the ratings.

##### Training of head nurses

**Fig. 2:**
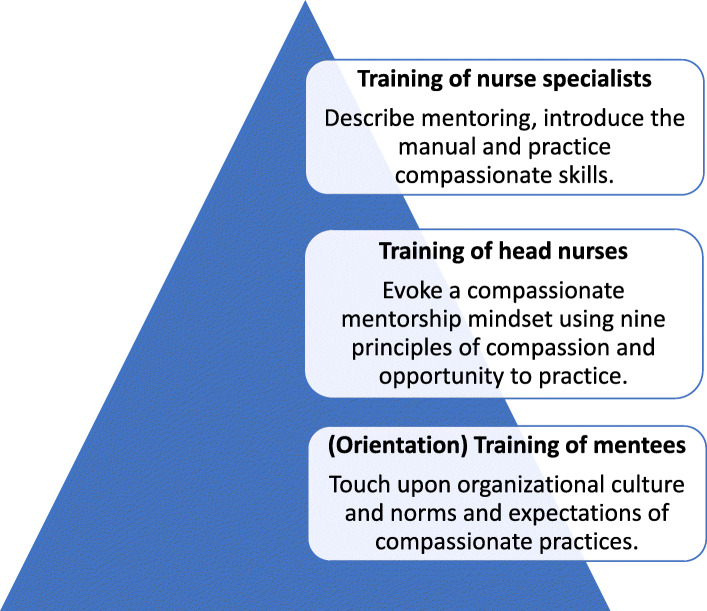
Training conducted for nursing specialists (supervisors/mentors of nurse mentors) and head nurses (nurse supervisors/mentors)

The head nurses also attended a similar 5-day workshop as their supervisors (nurse specialists) (Fig. 2). In the months to follow, special attention was dedicated to preparing the head nurses for training of their mentees (nurses). The Master Trainer and CiH lead from CfC Pakistan instated personal visits to the nursing specialists and head nurses and advised them on training styles and content. Mock presentations were then scheduled for meticulous feedback to strengthen the delivery of skills to the floor nurses. The mentorship trainings were envisioned to result in improved skills which will be beneficial in leading compassion trainings for the nursing staff. The expected outcomes of the training would be a number of head nurses certified by CfC to deliver trainings for compassionate skills.

##### Supervisory tools

**Fig. 3:**
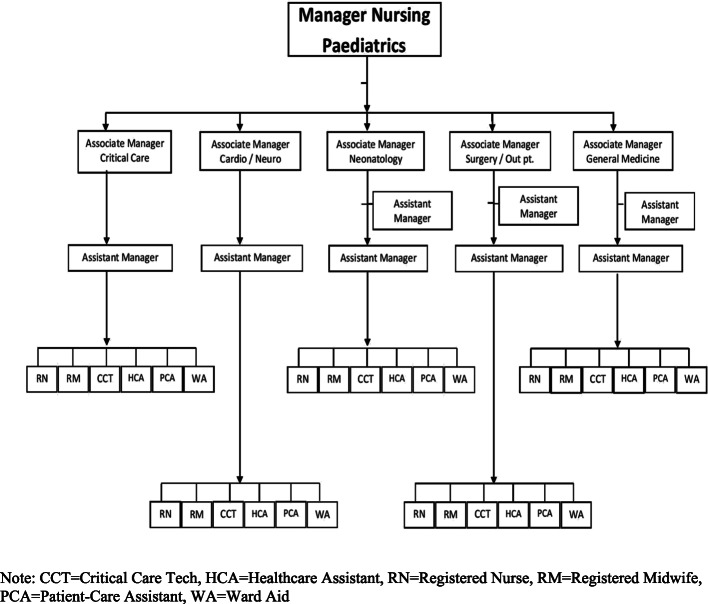
The revised organogram of nursing team

Supervisor-nurse ratio: The new organogram (Fig. 3) was more organized with a supervisor-nurse ratio decreased to 1:6. Each unit had its own nursing staff and supervisor. The nursing staff including registered nurse, registered midwife, healthcare assistant, patient care attendant, critical care tech and ward assistant, would report to ‘Assistant Manager’, who would further report to the Nurse Specialist (Associate Manager) of their respective units. Moreover, the new model implied that one Head Nurse (Assistant Manager) would observe the assigned nurse mentees on all skills (i.e., academic and administrative) while practicing compassionate mentorship. This would help the nurse mentee as he/she only would have to refer to one manager/mentor for help for his/her matters. It had also been decided that head nurses would also be given supervisory checklists to establish a structured feedback mechanism.

Manual: A manual was developed for the supervisors (head nurses) to provide an overview of the pediatric nursing mentorship process with all techniques and strategies for supervisors to mentor the floor nurses and standardize the process. The manual also outlined the two modes of mentorship communication: individual and group mentorship. The step-by-step guide suggested forming a flexible work plan with their supervisors (Nursing specialists’) consent and reviewing previous records. The manual was designed to respond to such improvements by providing practical situations and steps on how to apply mentorship in the most sustainable way.

Supervision checklist: As a part of implementing individual mentorship, it was envisioned that the nurses will be observed (sometimes unannounced as well), which will be followed by a collaborative discussion and constructive feedback on skills to improve (up to 3) and the ways to do it. To facilitate the process, a supervisory checklist was created which incorporated all the components related to their performance (Table 5). The checklist contained various elements that were objectified around the nine skills of compassion: for instance, nurses’ duties, such as regularity and punctuality were counted under ‘Integrity’, in the checklist. Each nurse was rated on a three-point Likert scale: with 0 being ‘no skill’ to 3 being ‘skill performance with excellency’. The checklist is available as supplementary material (Supplementary Table 5). Moving forward, nurses will be assessed by their respective head nurses using the checklist at least once every month. Following an observation, the total score for each section would be calculated and the nurse’s performance was allotted an overall rate. Once performance data for all nurses will be collected, it will be analyzed on a monthly basis and compared on various aspects: improvement in monthly performances of one mentee, comparative performance of mentees working under one mentor, and performance by different skills. Subsequently, a component named, ‘effective stress management’ was added in the checklist, to account for psychological health needs of the mentee.

**Table 5:** Items on the Supervisory Checklist mapped onto nine skills of compassion

Skills	Example
Mindfulness	Accurately assesses, timely identifies and appropriately responds to patient’s critical needs.
Courage	Shows Flexibility to accept various assignments as per need of unit/Service Line
Altruism	Guides new staff in provision of safe nursing care and in compliance of Hospital policies and procedures.
Gratitude	Demonstrates gratitude while interacting with the patient’s family, colleagues and immediate supervisors.
Integrity	Is regular and punctual with > 98% attendance and follows leave policy
Empathy	Demonstrates an understanding of patient needs and takes proper interventions accordingly
Self-Compassion	Keeps oneself well-groomed and maintains the uniform code at all times
Forgiveness	Demonstrates forgiveness in his/her behavior when dealing with patient and colleagues
Humility	Interacts with paediatric patients using play- based techniques

**Fig. 4:**
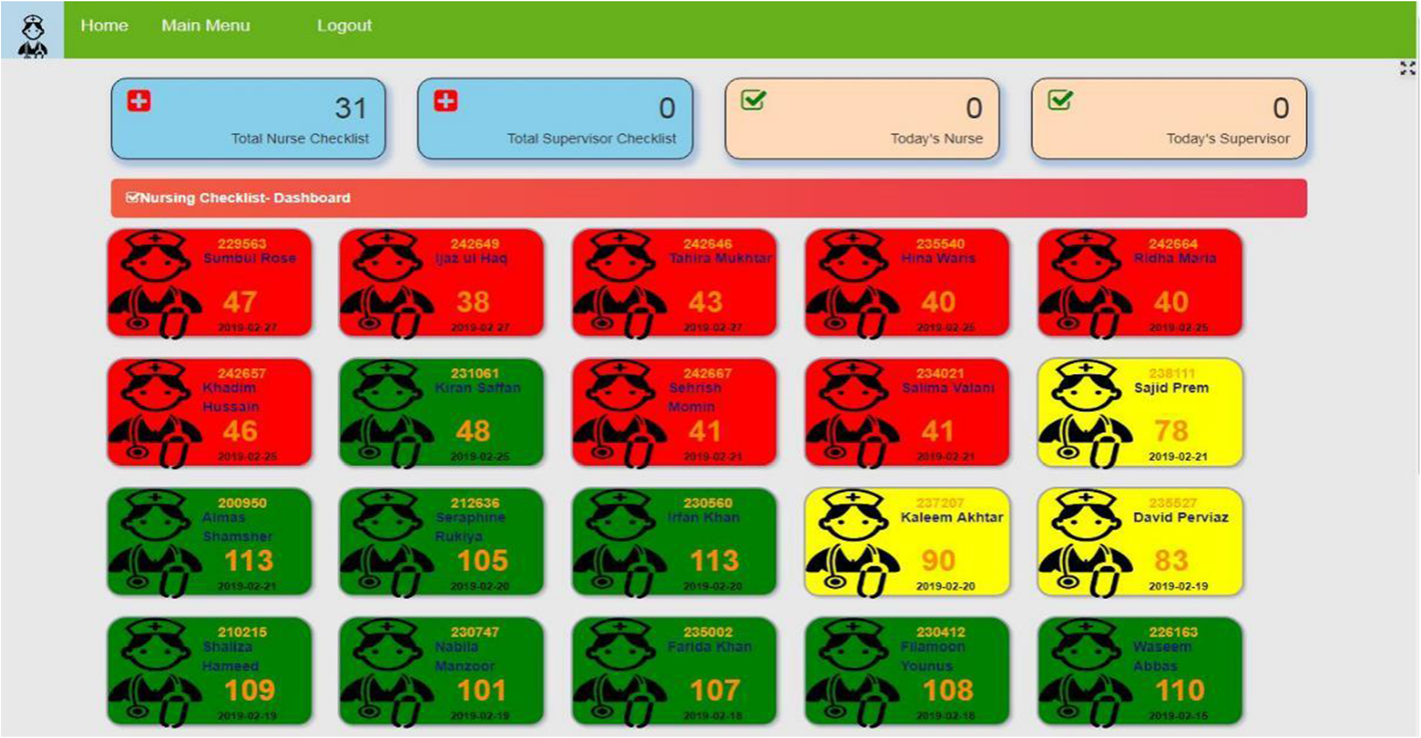
Electronic dashboard used to track each nurse mentee’s progress

**Fig. 5:**
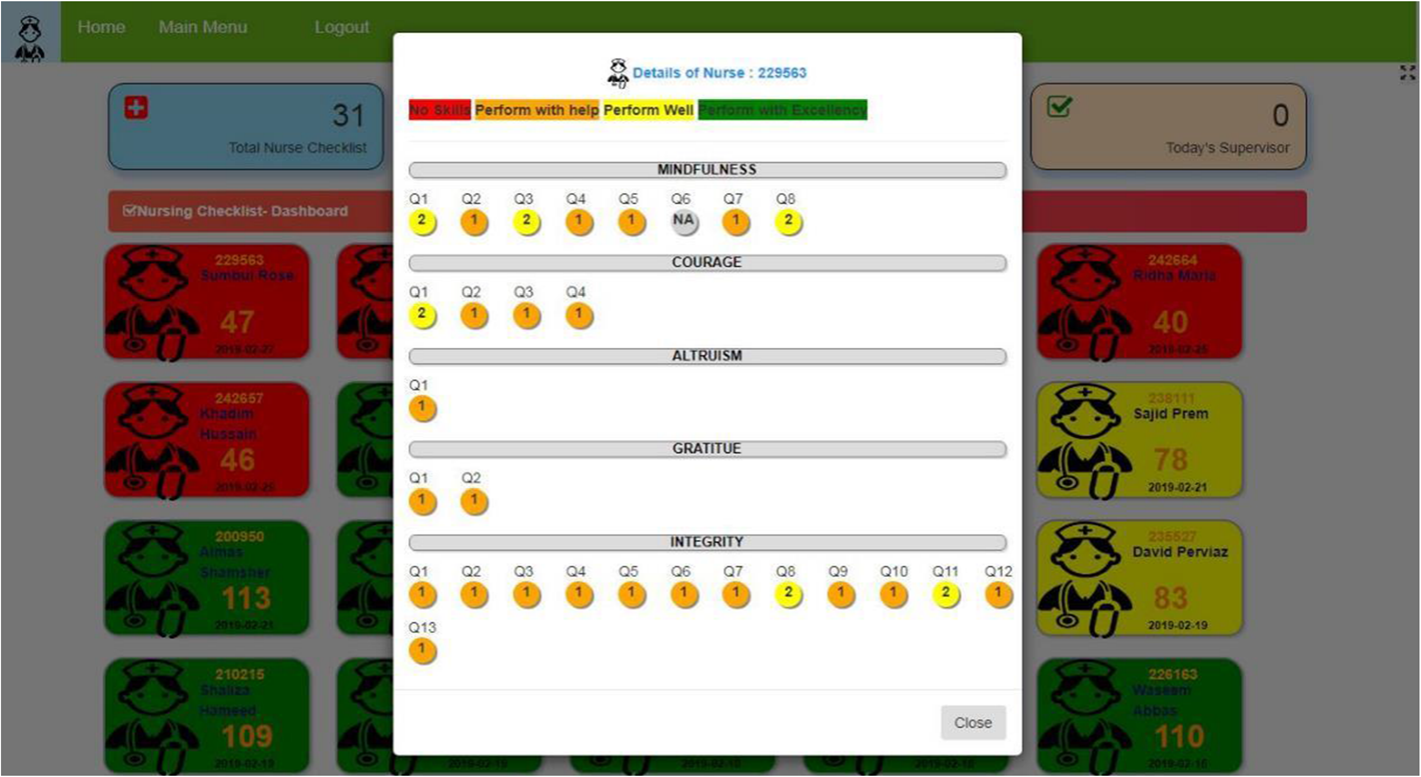
The dashboard tracking growth of the nurse mentee

Dashboard for data visualization: An electronic dashboard for head nurses was created and piloted in November 2019 to track each nurse’s progress visually to serve as a real-time supervision tool (Fig. 4). Thirty nurses and one head nurse participated in the pilot phase. Majority of the findings revolved around the need to simplify the electronic dashboard and data entry mechanism to make it more user-friendly. Observations were recorded by the head nurses using a tablet and data were synchronized to be displayed in the form of a nursing mentorship dashboard. On the dashboard, every nurse was color coded as red (overall performance score ≤ 39%), yellow (overall score 40–79%), or green (overall score ≥ 80%). The working team believed that the dashboard would be helpful for head nurses to compare performance of his/her nurses and was also to track their growth over a period of time (Fig. 5). Additional plans were to run a machine learning algorithm on the collected data to predict a nurses’ progress and skills that need to be augmented even before that nurse demonstrates any weakness in a component.

#### Promoting a culture of compassion

The call for sustainability and continued engagement resulted in another intervention for the head nurses (nurse supervisors); social media engagement. Numerous communication forums were utilized as pathways to create a culture of compassionate experience. This included recognition in town hall meetings, WhatsApp groups, morning huddles and a closed Facebook group. Moreover, appraisals were also planned as another approach to recognize the workforce performance. The nurse appraisal serves several purposes such as determining professional competence, enhancing staff development and motivating them toward higher achievement [57].

##### WhatsApp group

WhatsApp is known to be a famous application for smartphones that allows both one-to-one and group communication and is often used as a replacement to text messaging [58]. The WhatsApp accounts are based on an individual’s cell phone number and automatically generates the list of contacts. A WhatsApp group named ‘Nursing Mentorship Group’ was created to promote engagement among mentors through communication, coordination, feedback, and responsiveness. It was envisioned that when the head nurses are emotionally engaged through this continual knowledge sharing in the form of research, engaging content, real time examples of nurse-patient and mentor-mentee interaction and insightful discussions on the elements of compassion, they will successfully lead compassionate mentorship programme with the 413 nurses of the hospital. Social psychologists predict a link between social group and emotional connection, which may lead to cooperation and positive feelings [59]. To this end, the group was also an optimistic attempt to promote openness between nurse mentors and thus, strengthen peer relationships that have encompassing benefits for care providers and ultimately service recipients, i.e., the patients.

##### Promotion on the department Facebook Group

Compassionate values were further promoted through a closed Facebook group for the department [60]. This group was used among the service line team members and also served as one of the platforms to appreciate high performers among the team. The selection of ‘high performers’ was based on the appreciation received from patients on the institutional feedback form. Every member shared various informative messages regarding numerous topics that connected them to the organization and its patients for example, newsletters, insights from recent articles and useful tips that can be applied professionally etc. Moreover, achievements of units were also shared. This included the initiative named ‘Person of the Week’, where the employee was selected on the basis of nomination. Anyone on the Facebook group could suggest a nomination with the page administration team under a criterion shared on the group which included good performance and also showing compassionate behaviors. The name was announced on every Friday with a picture and paragraph written about the person’s strength. Out of 82 posts, a total of 18 were dedicated to the nursing team.

## Discussion

This paper presents a mentorship model hinged on compassion as an applied tool for growth and advancement of on-job nurses at a tertiary care hospital in Pakistan. Utilizing the theory of change model, interventions were designed across four broad domains: hiring staff with the right skills, strengthening supervision, providing nurses with on-job compassionate experience, and creating a culture that values human experience of compassion. An on-job mentorship programme was implemented with 35 nurses in the critical care department in a hospital in the USA for 6 months resulting in greater staff satisfaction and reduced turnover. The investigators followed a similar methodology to develop the programme but the mentors were external experts from universities and hospitals who were invited to share their experiences [61]. Another quasi-experimental study with the nurses and midwives in a Kenyan hospital found that targeted clinical mentorship improved their competencies contributing to a reduction in intrapartum fetal deaths. In this study too, the mentors were external experts working in their capacity as volunteers [62]. Issues with sustainability of an initiative is a concern especially when mentors are personnel from outside the organization.

The needs assessment with the patient survey indicated that the unique positioning of nurses caring for patients and collaborating with physicians made them extremely valuable stakeholders who must be placed at the epicenter of a compassionate healthcare movement. This is an observation from the health systems in high-income countries too where compassionate care has largely been associated with nursing among the healthcare providers [63]. In a system that assigned (almost unapologetically) compassion as a default virtue of all nurses, it was quite a proposition not only to create a programme implementing compassionate mentorship for the frontline staff but to predicate that compassionate experience can be created through formal and informal interactions.

While theoretically managers have been identified to have a key role in facilitating a compassionate environment, there are not enough implementation studies in the medical literature recognizing this role and thus laying the burden of the responsibility on the individual [64, 65]. It is in this ambition that we believe the value of our initiative lies i.e., a focus on enabling factors to ensure sustainability from the outset. The programme was postulated on the vision that care attributes begin reflecting in the attitude and actions of the novice nurses when supervisors as mentors become the role model of compassionate values, thus, creating better human experience spiraling into improved patient experience.

The majority of nurses had issues of being overburdened due to workload, in comparison to attaining a lower salary. Hartzband and Groopman indicate a similar circumstance and further reveal that monetary incentives are not a solution for burnout [66]. In fact, according to a recent survey, half of the doctors including millennials who have the lowest salaries, were willing to give up at least minimum $20,000 of their annual incomes, in exchange for reduced working hours [67]. Burnout can be eliminated from the caregivers’ routine through incorporating three main pillars: autonomy, competence and relatedness, in the healthcare profession [66]. The current study, at some level, utilizes these pillars in the on-job mentorship program to promote a conducive mentor-mentee relation, however its primary focus is to apply a compassionate framework to reduce burnout and compassion fatigue.

The study addresses not only the need of eliminating negative perceptions related to work but also for improvements in the existing supervision and mentoring process. The amalgamation of compassion literacy in the guided framework of the intervention model along with a basic training for mentorship can have a positive effect on mentor-mentee relationships through enhanced engagement which ultimately improves employee experience in the healthcare organizations. The study emphasizes immense importance on the role of managers as mentors who are crucial for the professional growth and advancement of on-job nurses in clinical roles- an important factor to ensure employee engagement. Hence, training should be provided to enable them to learn new skills that align with their supervisory roles i.e., being objective, concise, skill driven and being able to utilize systemic analyses to overcome any ongoing nursing practice challenges with their mentee team.

Preliminary evidence from participant feedback, both from the first set of workshop trainings for mentors and subsequent mentee trainings, concurs that a compassionate mindset can be cultivated by learning about compassion. However, the programme is in its infancy stage and the impact can be assessed a few years later. For the required impact to occur, a focus on shaping the culture for compassionate care with multiple interventions has to continue besides training for skills including communication of the value placed on these skills and measurement of the skills in the organization to keeo the staff motivated [68].

Having a balanced team with the right skills and expertise emerging from a conceptual framework is vital to effective intervention design [35]. The AKUH team hence made an effort to find a partner with experience of implementing programmes around compassion. CfC Pakistan’s Compassionate Skills programme afforded the much-needed objective to form compassion – a deceptively simple concept, often disregarded for difficult measurability and skill transference. The skills were broken down into nine different but interconnected elements, combined with the Master Trainer’s expansive repertoire of engagement activities and exercises, invoked relativity and emotionality in the participants who later wrote testimonials for the notable impact on their self-views, both as care-providers and supervisors. The discourse on compassion was strengthened and reinforced, complimenting the changing culture at children’s hospital, with subsequent visits by the CiH lead who prepared routine supervisors/managers as nurse mentors for the next rollout of trainings. The partnership also yielded the opportunity to engage nurses (rather informally) on WhatsApp wherein a compassionate skill was discussed each week with an emphasis on lived work experiences around it. Expression of gratitude and acknowledgment of colleagues improved on this informal forum.

The collaboration held merit because it favored a shared learning experience for stakeholders at both organizations. A solid evidence for the efficacy of this partnership is the Nurse Performance Checklist – technical evaluation protocols for nurses fused with elements of compassion. Success of the partnership is also attributed to the well-informed progression planning by the Theory of Change team that created a guided step-by-step framework. Improved wellness, emotional security, interpersonal and intrapersonal connectedness, engagement, and eventually better patient experience are few of the many encouraging outcomes of compassionate mentorship, serving the vision of both partners, i.e., compassionate healthcare. An overwhelming caseload on the nurses and encountering massive deaths, due to the prevalence of global pandemic have caused a decline in mental well-being. Moreover, staff from a high-income countries like publicly funded healthcare system in Wales, have raised concerns due to bullying stating that they (nurses) were not allowed time off for appointments and to use annual leaves as they had wished. Along with this, completion of tasks was seen as more important for managers than the nurses’ well-being [69].

According to a global study in 2020, almost 40% of employees from more than ten industries, complained that their companies have not asked them how they were doing since the pandemic [70]. Such instances demand for instigating a compassionate framework in organizations to sustain workers’ mental health. Moreover, when asked to indicate their preferences for sharing mental health concerns with the participants chose peers and managers. Hence, a recommendation for future research could be to focus on the application of managers as mentorship guides in other organizations.

Absence of Standard Operating Procedures (SOP) for partnership was a potential limitation in the study. While learnings were shared, and collaboration transpired consultatively, it may be important to develop SOPs as partnership deepens and more actors are involved to achieve the set vision. Lack of planned activities over time, including refresher trainings for the nurse mentors due to their prolonged on-job hours, could also be a potential limitation. However, the likely effects were reconciled through follow-up preparatory sessions and mock presentations with the master trainer. WhatsApp group also served to reinforce learnings from the workshops. Furthermore, there was no checklist to evaluate the performance of the nurse managers (supervisor of the nurse specialists). The current intervention model study does not state their accountability despite their appraisal being done by SLC and the CEO. This aspect is a consideration for the future.

## Conclusion

Nursing communication is known to be a primary driver of family experience in pediatric healthcare. Enhancing their abilities by imparting a compassionate experience using on-job mentorship, enables them to reciprocate compassionate care to their patients. The current study identified and trained supervisors as a feasible and effective strategy to provide compassionate experience to nursing staff as a strategy to deal with their pain points at a systemic level in a tertiary care center in a low-to middle -income country. The goal of sustainability can be achieved by aligning mentorship responsibilities as a part of job description and institutional performance management system, when designing mentorship programmes.

## Supplementary Information

**Additional file 1: Tables.** Core Team Members, Working Team of the Head Nurses for Training Workshops, and Aims, Methods used and Key Activities of the Training Sessions, Supervision. **Figures** for ‘Old Organogram of the Children’s Hospital Service Line’, Job Description of Nursing Associate Manager’ and Nursing Assistant Manager.

## Data Availability

The datasets used and/or analyzed during the current study are available from the corresponding author on reasonable request.

## Abbreviations

AKU, K: Aga Khan University (Karachi)
ToC: Theory of Change
CfC: Charter for Compassion
RACI: Responsible, Accountable, Consulted and Informed
NES: Nursing Education Services
RNs: Registered Nurses
HCAHPS: Hospital Consumer Assessment of Healthcare Providers and Systems
CNI: Clinical Nurse Instructor
CiH: Compassion in Healthcare
