# Supplementary material for: Development of an on-job mentorship programme to improve nursing experience for enhanced patient experience of compassionate care
Source: BMC Nurs. 2021 Sep 18;20:175. doi: 10.1186/s12912-021-00682-4 (PMC8449216; doi:10.1186/s12912-021-00682-4)
Supplement: Supplementary file 1 — Additional file 1: Tables. Core Team Members, Working Team of the Head Nurses for Training Workshops, and Aims, Methods used and Key Activities of the Training Sessions, Supervision. Figures for ‘Old Organogram of the Children’s Hospital Service Line’, Job Description of Nursing Associate Manager’ and Nursing Assistant Manager. [file 12912_2021_682_MOESM1_ESM.docx]

Table 1: Core team members, their expertise and years of experience

| **Name** | **Role** | **Domain expertise** | **Years of Experience** |
| --- | --- | --- | --- |
| AZ | Compassion specialist | Implementing compassionate practices in education sector | 5 |
| BS | Service Line Chief | Paediatric cardiology, quality improvement and health outcome measures. | 15 |
| MR | Director Patient Experience of Care | Behaviour scientist with experience in implementing interaction-based interventions | 10 |
| NL | Nurse Manager | Nursing administration and education | 19 |
| NS | Nurse Specialist | Nursing administration and education | 11 |

Table 2: Working Team of the Head Nurses for training workshops

| **Name** | **Expertise** | **Years of Experience** |
| --- | --- | --- |
| AR | Nursing Education | 7 |
| FS | Nursing Administration | 8 |
| GE | Nursing Administration and Education | 9.5 |
| JS | Nursing Education | 9 |
| RI | Nursing Administration and Education | 10 |
| SA | Nursing Administration | 9 |
| SD | Nursing Administration and Education | 15 |
| SH | Nursing Administration | 6 |
| YH | Nursing Administration and Education | 24 |
| ZAM | Nursing Education | 8 |
| ZA | Nursing Administration | 5 |

Table 3: The aims, methods used and key activities of the training sessions

| **Session** | **Aims** | **Key Activities** |
| --- | --- | --- |
| Introduction to Supervision | Understand the roles and responsibilities associated with mentorship, explore critical skills and implement it. | Describe a supportive supervisor and make transition in understanding of their roles from coordination and management to supervising. |
| Effective Communication | Emphasized on the importance of effective communication skills and the common understanding formed. ‘Care Feedback’ model was introduced as a strategy of providing constructive feedback. | Supervisors (mentors) to identify their communication styles: verbal, non-verbal, and written, and its impact on being an effective supervisor. |
| Learning to Observe | Understand the importance of observation skills, which areas to observe, become familiar with taxonomy reflections, and practice written communication. | An opportunity to practice constructive feedback, but also demonstrate basic understanding of reflection. |
| Problem Solving | Understand basic principles of data analysis and strategizing. | Learn the use of checklists to identify problems, familiarize supervisors with participatory problem solving, prioritize actions, and learn how to use observations in order to build the team’s capacity |
| Coaching | Teach the mentors on how to coach nurses with focus on utilizing the respective skills in numerous situations. | Help supervisors mentors understand coaching, building capacity and scaffolding, enabling and empowering them. |

Table 4: Supervision Checklist for Supervisors (Head Nurses)

| **Supervision Checklist for Supervisors** | |
| --- | --- |
| **Instruction:** This form will be used by the Senior supervisor to observe the nurses supervisors. Mark N/A if item is not applicable.    Rate the item on scale of 0-3  0= Weak skill, 1 = Performs with help, 2 = Performs well, 3 Performs with excellence  At the end of supervision conduct feedback session and share the scores with staff | |
| **Name of Senior Supervisor (Observer)** | |
| **Name of Supervisor** | |
| **Employee number** | |
|  | |
|  | **Rating** |
| **Date:** |  |
|  |  |
| **Organization skills** |  |
| 1. Punctuality |  |
| 1. Regularity |  |
| 1. Follows proper process for leaves |  |
| 1. Organize logistics (equipment, supplies, medicines , forms) |  |
| 1. Priorities the tasks effectively |  |
| **Technical skills** |  |
| 1. Observes the staff effectively |  |
| 1. Provides on job coaching in effective way |  |
| 1. Step in and provide support to staff appropriately when needed |  |
| 1. Conducts structured feedback session |  |
| 1. Share the observations with staff and disclose the supervisory checklist. |  |
| 1. Gives opportunity to staff for self-reflection and help in the process. |  |
| 1. Focus on both strengths and weaknesses |  |
| 1. Suggests appropriate strategies for improvement. |  |
| **Education session** |  |
| 1. Plans education sessions based on training need assessment. |  |
| 1. Conducts sessions effectively |  |
| 1. Encourages participation/ engagement from group during sessions |  |
| 1. Begins and finishes sessions on time |  |
| 1. Maintains pace during sessions |  |
| 1. Summarizes key content at the end of session |  |
| **Communication skills** | |
| 1. Effective communication with staff/mentee   · Smile  · Friendly tone  · Eye contact  · Appropriate facial expression |  |
| 1. Encourages and praise the staff |  |
| 1. Maintain privacy of staff performance |  |
| **Coordination skills** | |
| 1. Team work |  |
| 1. Coordination with fellow supervisors |  |
| 1. Coordination with Senior supervisor |  |
| 1. Interpersonal Problem solving |  |
| 1. Conduct and discipline |  |
| 1. Positive attitudes |  |
| Total Score (%) |  |

Table 5: Supervision Checklists for Nurses

| **Supervision Checklist for Nurses** | |
| --- | --- |
| **Instruction:** This form will be used by nurses’ immediate supervisor (head nurses and instructors) Mark N/A if item is not applicable.    Rate the item on scale of 0-3  0= Weak skill, 1 = Performs with help, 2 = Performs well, 3 Performs with excellence  At the end of supervision conduct feedback session and share the scores with staff | |
| **Name of Supervisor** | |
| **Name of Staff** | |
| **Employee number** | |
|  | ***Rating*** |
| **Date:** |  |
| **MINDFULNESS** |  |
| 1. Safe in patient care |  |
| 1. Safe in medication administration |  |
| 1. Accurate assessment, timely identification and appropriate response to patient’s critical needs. |  |
| 1. Maintains and ensures availability of required supplies in unit at the beginning of each shift |  |
| 1. Is aware of reportable patients in the unit |  |
| 1. Responds to patient call bell immediately |  |
| 1. Assesses the patient environment for safety and proper functioning of equipment and takes action as appropriate. Reports to head nurse/ team leader as required. |  |
| 1. Comply with International Patient Safety Goals while caring for the patients. |  |
| **COURAGE** |  |
| 1. Flexibility to accept various assignments as per need of unit/Service Line |  |
| 1. Reports incidents on timely basis |  |
| 1. Accepts error and take criticism positively |  |
| 1. Participates in combined rounds and discusses patient care plans with physicians on their rounds. |  |
| **ALTRUISM** | |
| 1. Guides new staff in provision of safe nursing care and in compliance of Hospital policies and procedures |  |
| **GRATITUDE** | |
| 1. Demonstrates gratitude while interaction with patient’s family |  |
| 1. Demonstrates gratitude while interaction with colleagues and immediate supervisors |  |
| **INTEGRITY** | |
| 1. Regularity and punctuality with >98% attendance and follows leave policy |  |
| 1. Follows institutional policy for handling patient’s valuables |  |
| 1. Maintains Confidentiality about patients |  |
| 1. Maintains Confidentiality about colleagues |  |
| 1. Timely and appropriate documentation |  |
| 1. Attends training sessions (when nominated) |  |
| 1. Demonstrates safe infection control practices including hand hygiene |  |
| 1. Age appropriate and gender sensitive care |  |
| 1. Priorities the tasks effectively and keeps assigned patient area well organized and clean |  |
| 1. Follows admission/transfer/discharge process according to hospital protocol |  |
| 1. Organize MMU processes of assigned patients |  |
| 1. Efficiently utilize feed handling process |  |
| 1. Demonstrate accountability towards special assignments like crash cart, floor stock, CSSD, narcotics, etc as assigned by supervisor |  |
| **EMPATHY** | |
| 1. Confidentiality about patients |  |
| 1. Confidentiality about colleagues |  |
| 1. Understanding of patient needs and takes proper interventions accordingly |  |
| 1. Gentle handling of patients |  |
| **SELF-COMPASSION** | |
| 1. Updates own skills by attending certification and recertification courses as per credentialing requirement |  |
| 1. Keeps oneself well-groomed and maintains the uniform code at all times |  |
| **FORGIVENESS** | |
| 1. Demonstrates forgiveness in his/her behavior towards dealing with patient and colleagues |  |
| **HUMILITY** | |
| 1. Keep play based interaction with patients. |  |
| 1. Effective communication with patients and families:   · Smile  · Friendly tone  · Eye contact  · Appropriate facial expression |  |

Figure 1: Organogram of the Children’s Hospital Service Line

*
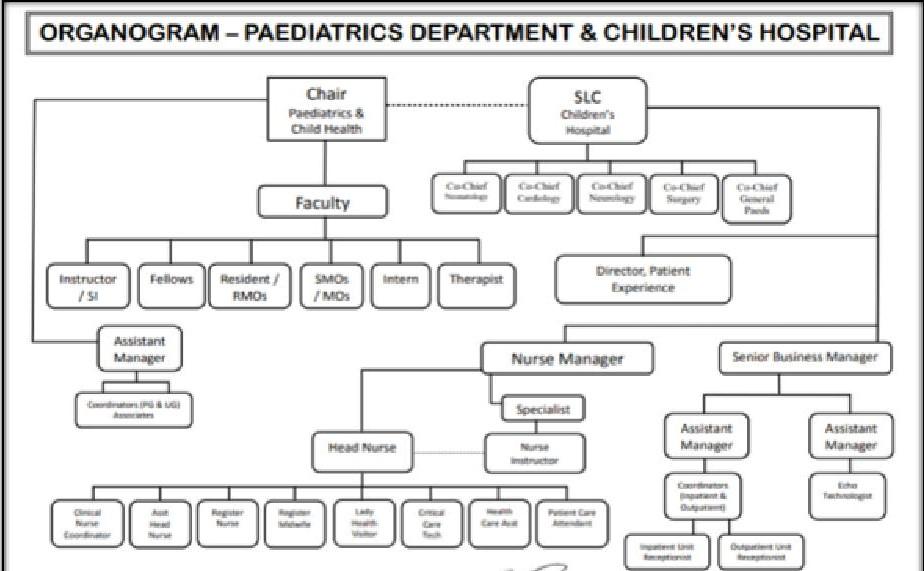
*

Figure 2: Job Description of Nursing Associate Manager

*
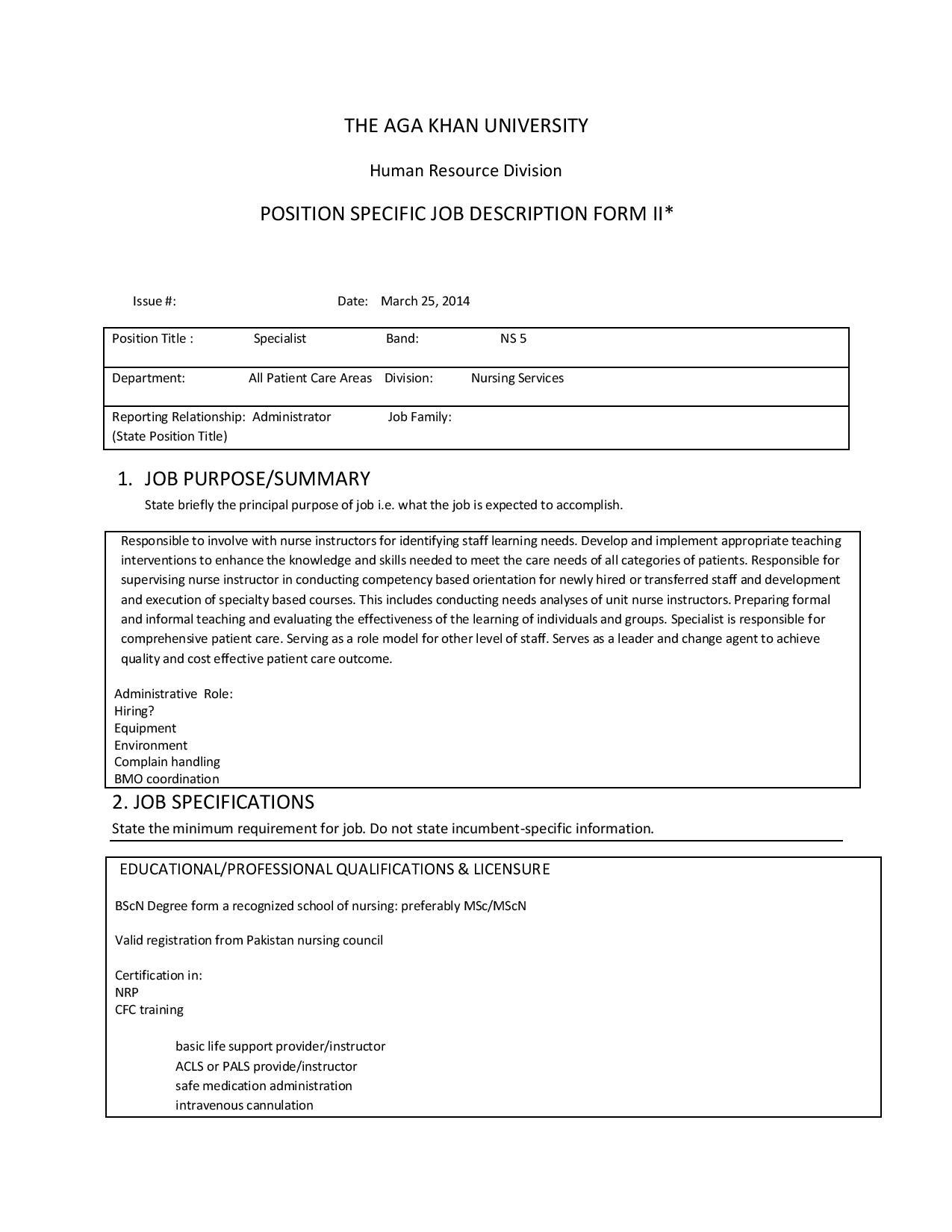
*

*
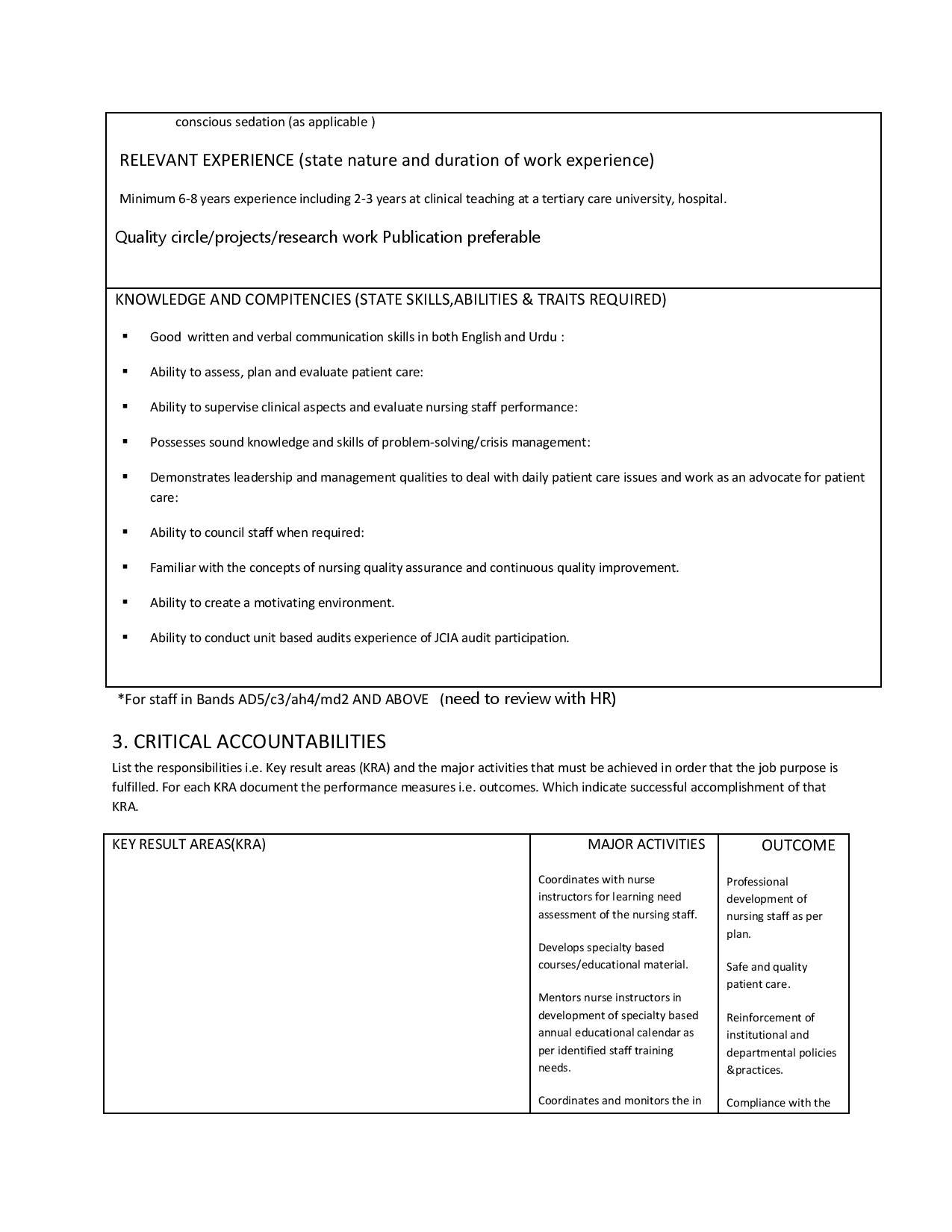
*

*
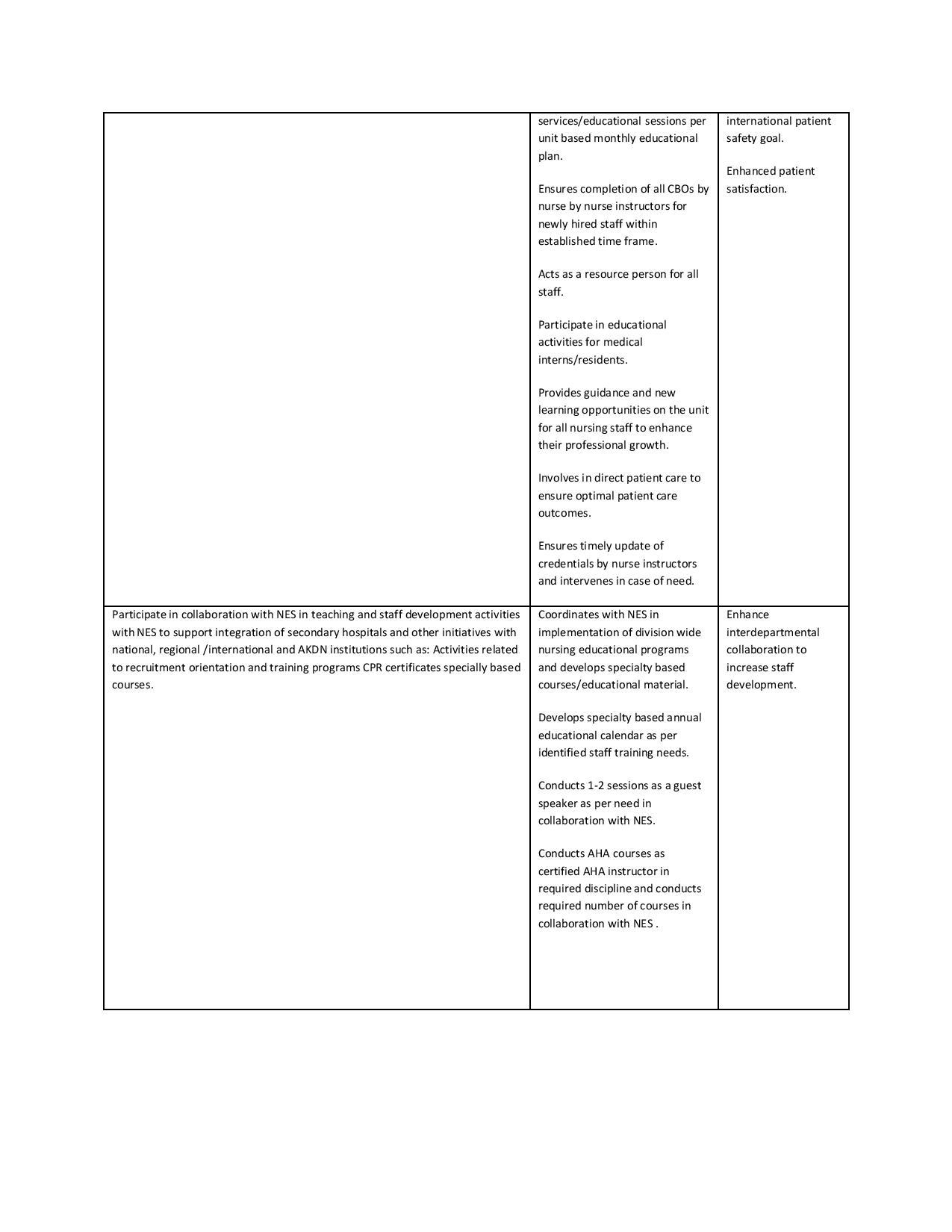
*

*
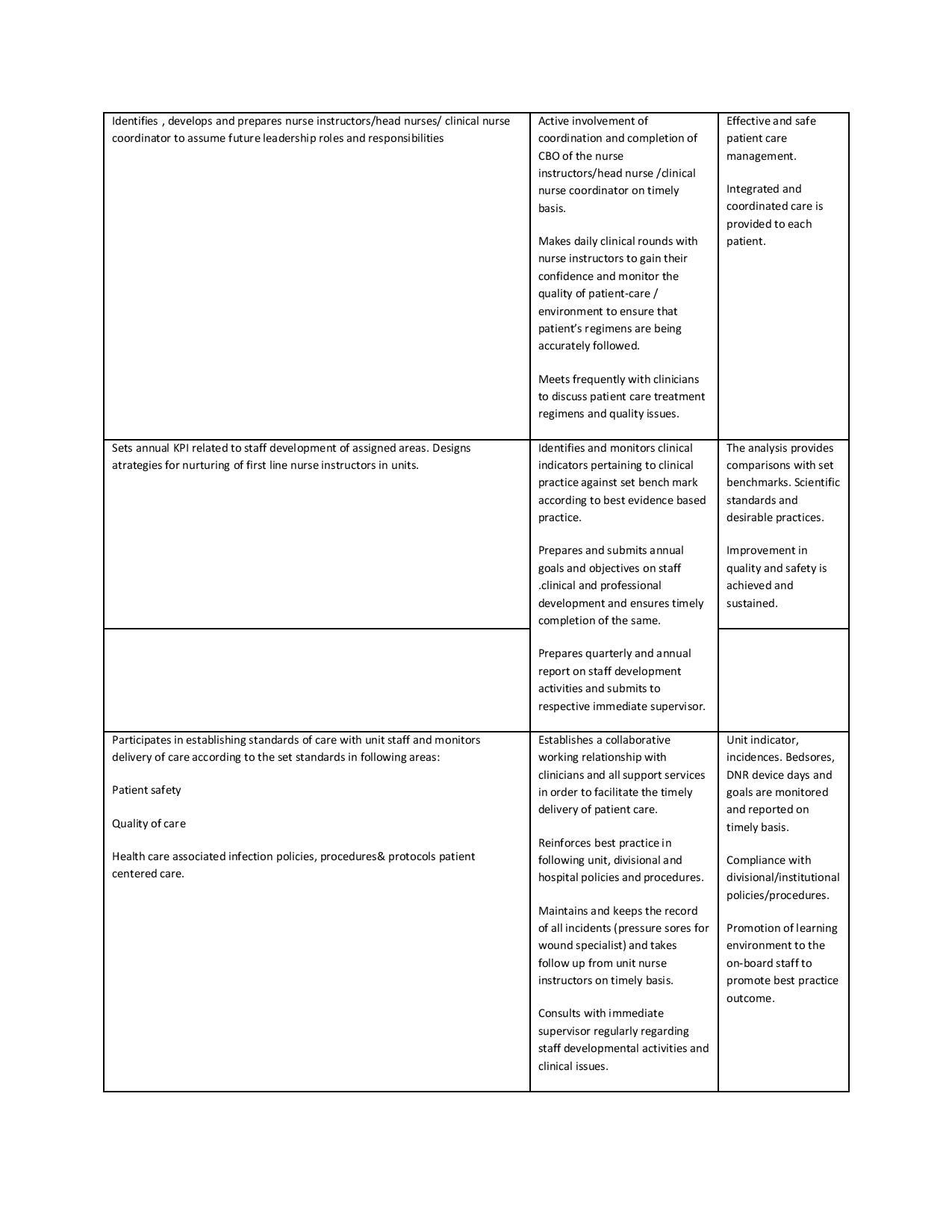

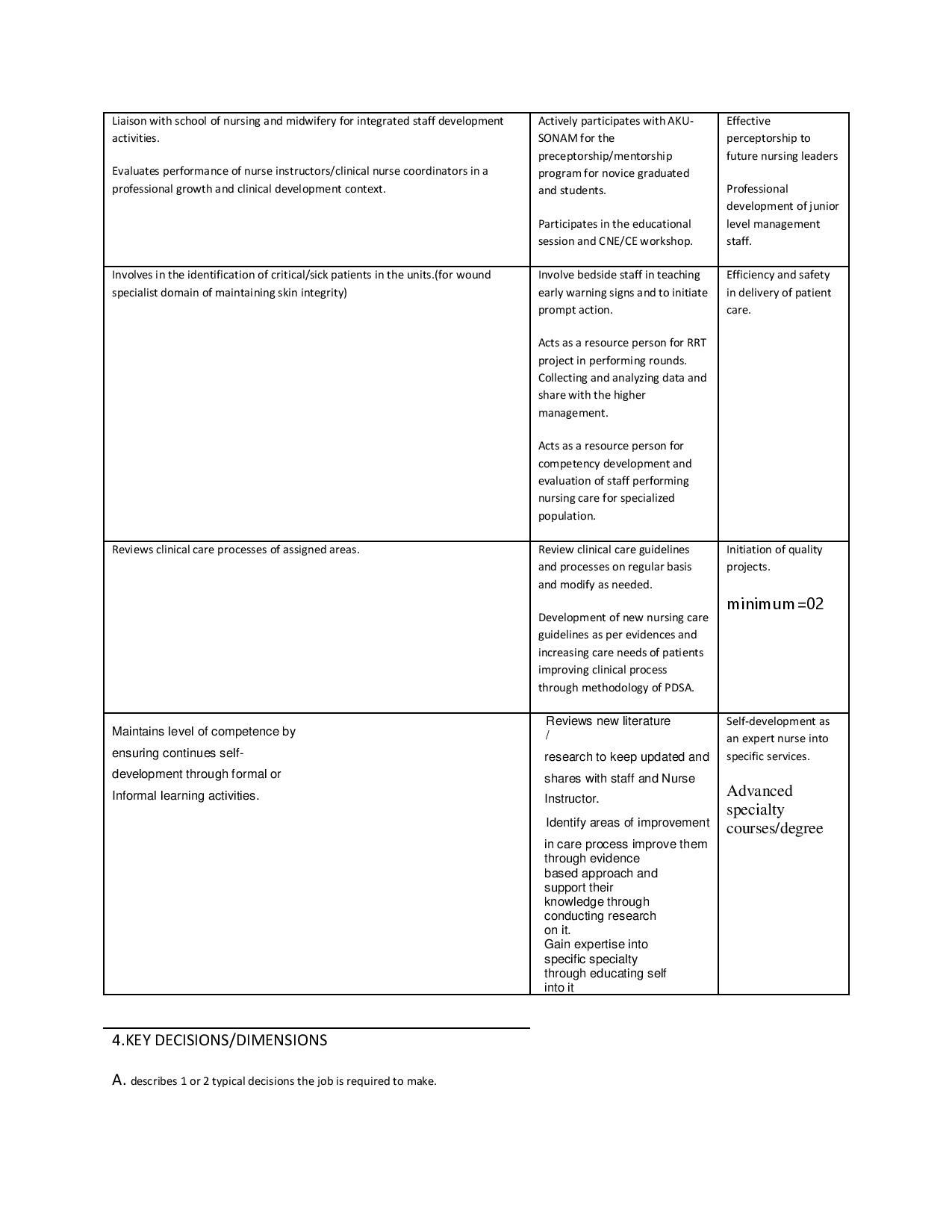
*

*
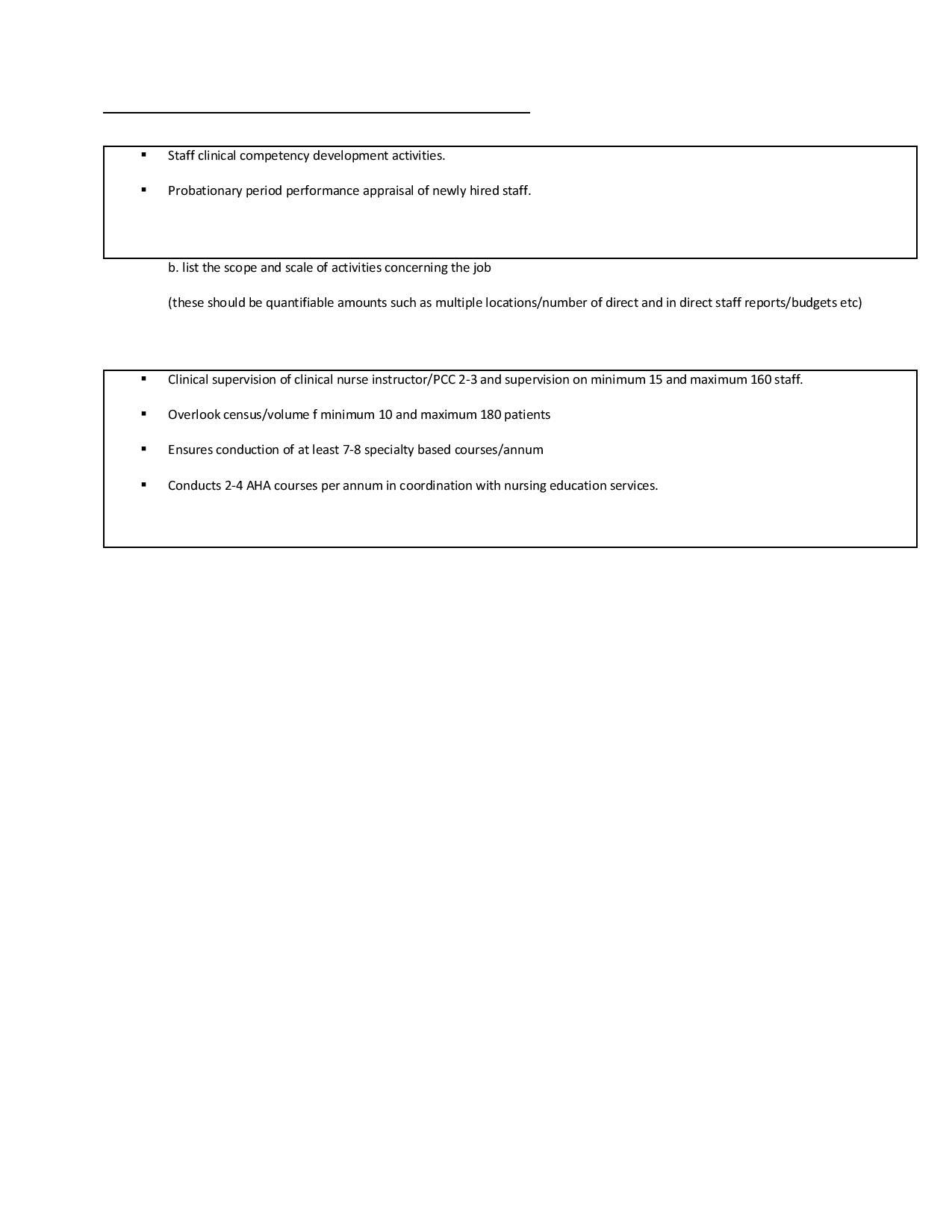
*

Figure 3: Job Description of Nursing Assistant Manager

*
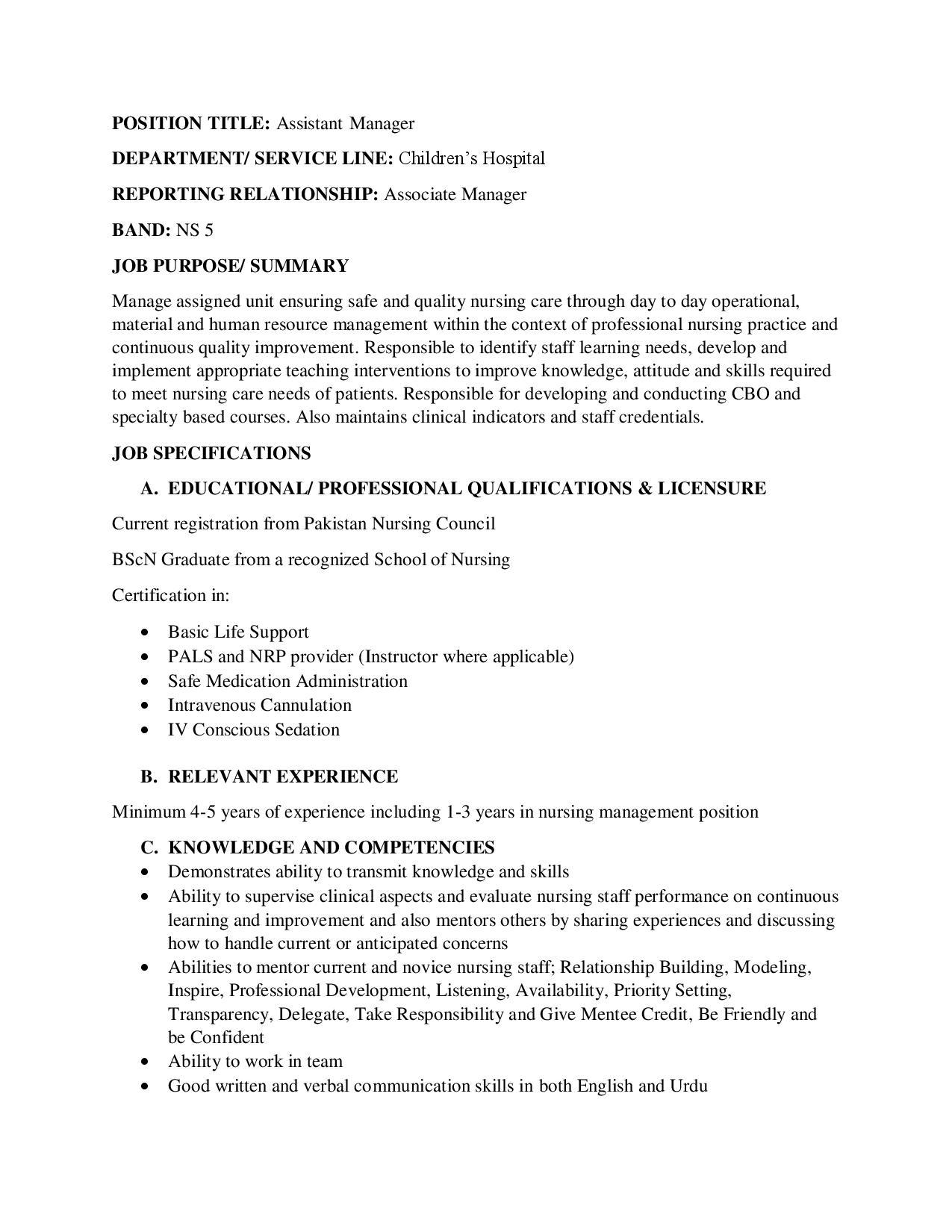
*

*
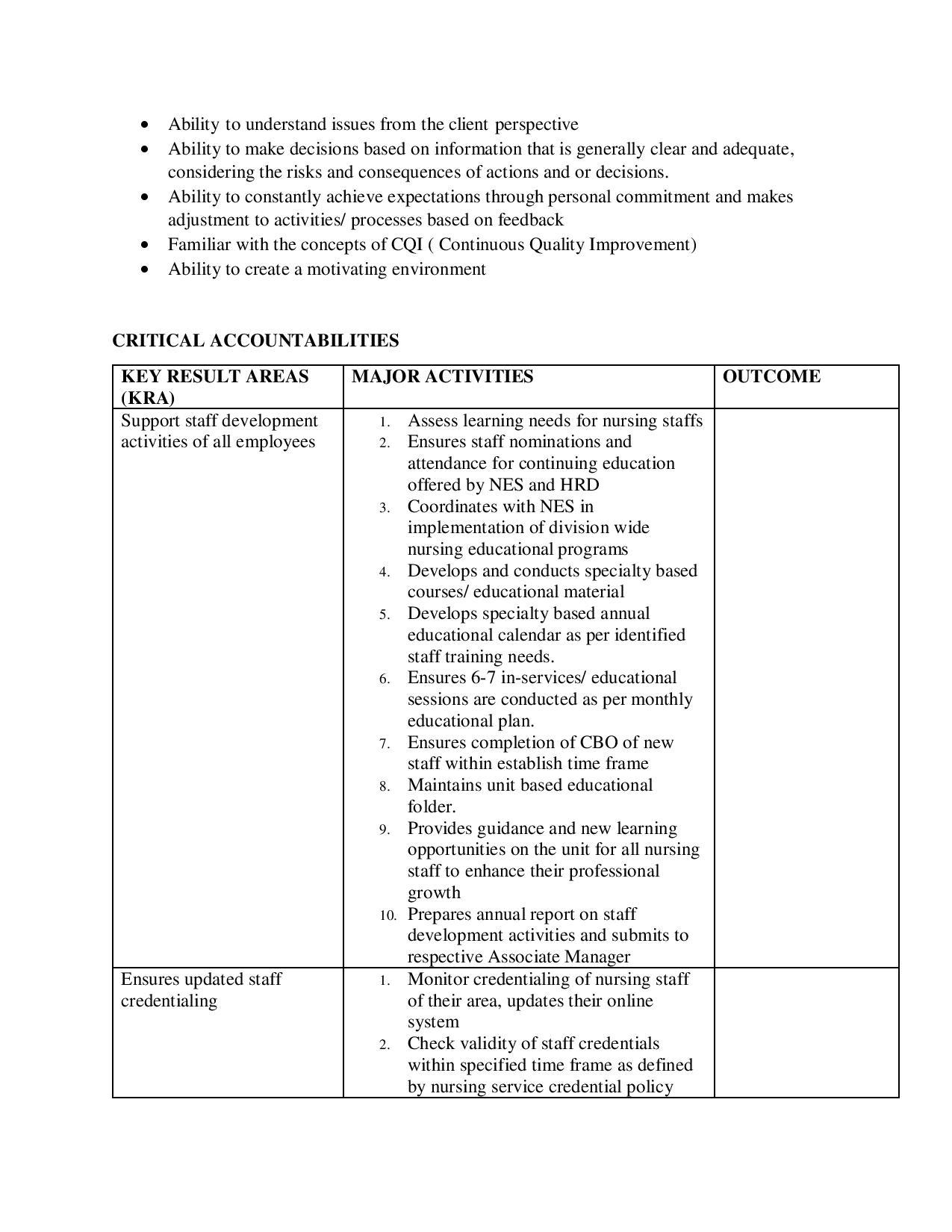
*

*
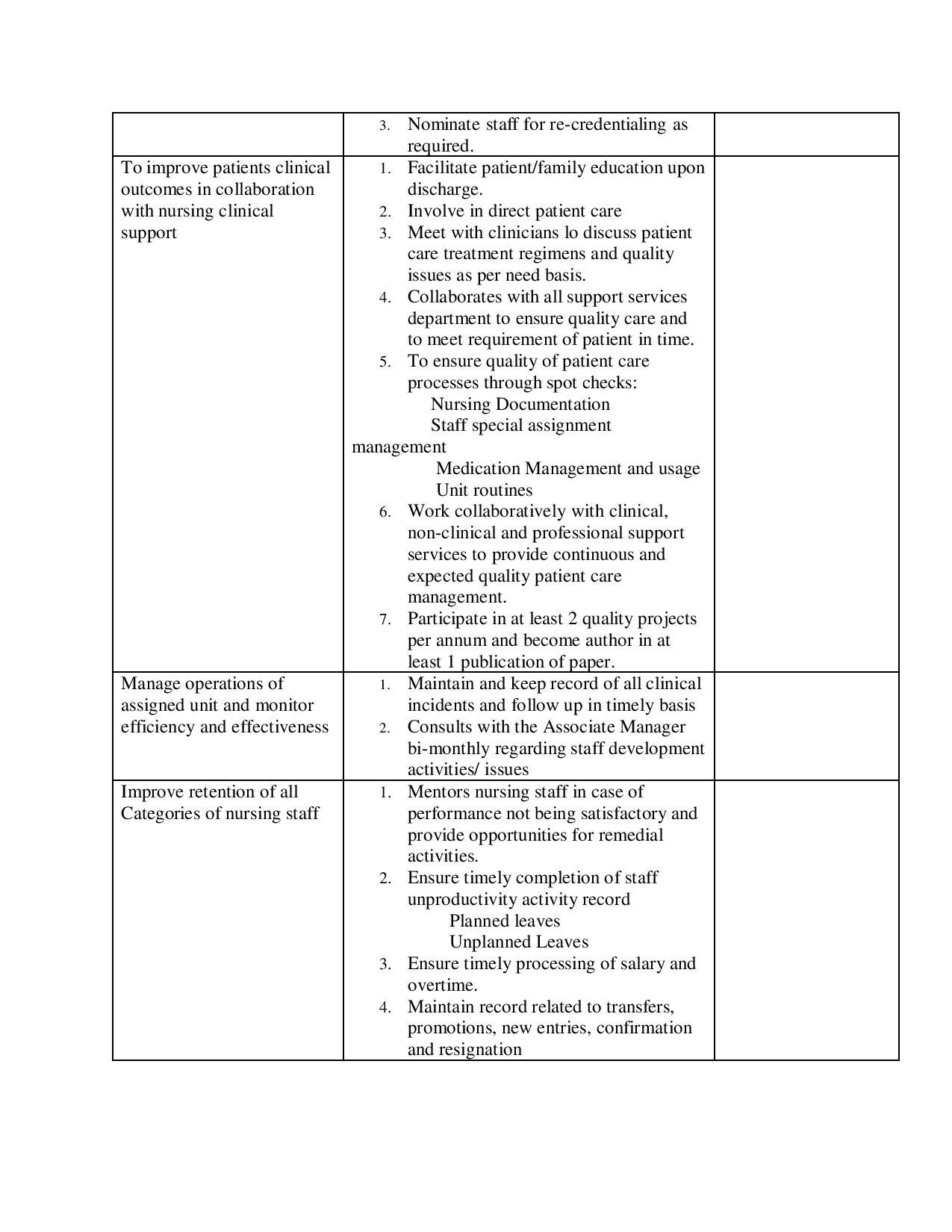
*
